# Establishing forward mixing model to mass transfer overview in multi-impeller agitated column for rare Earth extraction process

**DOI:** 10.1038/s41598-024-52961-0

**Published:** 2024-02-28

**Authors:** Rezvan Torkaman, Mohammad Reza Aboudzadeh Rovais, Mehdi Asadollahzadeh, Meisam Torab-Mostaedi, Mojtaba Saremi

**Affiliations:** 1 Nuclear Fuel Cycle Research School, Nuclear Science and Technology Research Institute, P.O. Box: 11365-8486, Tehran, Iran; 2 Radiation Application Research School, Nuclear Science and Technology Research Institute, P.O. Box: 11365-8486, Tehran, Iran; 3 Energy Engineering and Physics Department, Amirkabir University of Technology, P.O. Box: 15875-4413, Tehran, Iran

**Keywords:** Mathematics and computing, Chemical engineering

## Abstract

The current study develops comprehensive mass transfer models to optimize the rare earth extraction. A plug flow, axial dispersion, backflow, forward mixing-based mass transfer model was created and solved numerically using the fitting technique. The investigated process is a multi-impeller agitated column designed to provide proper contact between organic and aqueous phases to extract rare-earth ions. Taking Sm(III)–Gd(III) separation as an application case, extraction efficiency in the agitation speed of 200 rpm was obtained equal to 95.14%, 76.67% by this column for Gd(III), and Sm(III) ions, respectively. The model's findings were compared with experimental data, and a significant agreement was achieved with the forward mixing model. The results indicated that the high agitation speed is beneficial to increasing the interfacial area while reducing the mass-transfer coefficient. On the contrary, the circulation within the larger droplet improves the transfer of mass, albeit at the expense of reducing the interfacial area. The results showed that the drop size distribution is a crucial factor as the droplet sizes significantly affect the droplet mass transfer. The mathematical models’ values of E_c_ for mass transfer parameters showed that the operational variables significantly affect the mass transfer rate and can cause deviations from the ideal flow path. A reasonable and appropriate estimation of the organic-side volumetric overall mass transfer coefficient was provided, which can be applied to this contactor’s design and scale-up.

## Introduction

A multi-impeller agitated column, a typical extraction process equipment, achieves a high extraction efficiency using an increased agitation speed^1,2^. Numerous liquid droplets were created, exhibiting excellent micro-mixing, and mass transfer performance^3,4^. The agitation extraction columns have received attention worldwide owing to the advantages of high efficiency, and safe operation^5^. This equipment has been widely used in metallurgical processes to purify the leaching solution^6,7^. Extracting rare earth metals is extensive, and different minerals and their various behaviors are the challenges for purification. The refining process for the production of rare-earth metals with strategic applications is critical, and part of the vital knowledge depends on the ore structure^8^, leaching parameters^9^, organic extractants, and mass transfer equipment^10^. The development of extractive solvents in separating these metals has led to various processes with specific advantages. Various extractants have been developed with a green approach and reduced environmental impact^11,12^. The knowledge of the chemistry of solvents has dramatically expanded in producing rare-earth metals. However, there has been limited research on the analysis of specific equipment, and purification is carried out using mixer-settlers as a form of industrial machinery^13^. In order to enhance the process, researchers have created several laboratory-scale devices like membranes and micro-channels to extract and separate rare earth metals^14,15^. But, the research on a pilot scale is limited to a small number of studies.

In these extractors, the hydrodynamics of the flow is very important, and the distribution of droplets and their size, the dispersed phase holdup, and the slip velocity of the phases cause the flow dynamics to be achieved by the desired conditions^16–20^. Mass transfer is another examination hotspot other than the hydrodynamic characteristics for the scale-up of the extractor, which is advantageous for the intellectual upgrade of the scale-up impact and better comprehension of the scale-up process^21^.

Asadollahzadeh et al. measured the mass transfer performance of the Oldshue-Rushton column using standard chemical systems. They found that volumetric overall mass transfer coefficients were similar under equal energy dissipation rates. However, changes in operating conditions and physical properties of the systems have an essential effect on the interfacial area, and it is associated with variation in mass transfer in different systems^22,23^. Kumar and Hartland studied organic-aqueous mass transfer rates of various columns, including pulsed plate, RDC, Kühni, and Karr Columns. They proposed the correction factors for determining mass transfer coefficients from single drops and liquid–liquid extraction column data^24^. The study of mass transfer in the scale-up extractor will play a significant role in an industrial application in the future. The utilized method to measure mass transfer coefficients on both sides varies from empirical to theoretical, first-principles, and mechanistic models^25^. Plug flow, backflow, axial dispersion models are essential to describe mass transfer. However, the primary concern revolves around the lack of consistency in droplet size within the column. This inconsistency hinders the establishment of an ideal flow within the column, leading to a substantial error in determining mass transfer coefficients using these models. In the forward mixing model, droplet size distributions and droplet velocities appear, which play an essential role in optimizing the mass transfer coefficients^26^. Studies on mass transfer in multi-impeller agitation columns are available in the literature, emphasizing and investigating the organic-side mass transfer coefficient (K_od×_a) through mechanistic or empirical methods^27,28^.

In this work, mass transfer, including effective interfacial area (a) and dispersed-side overall volume mass transfer coefficient of the pilot-scale multi-impeller column, was investigated using the extraction and separation of samarium and gadolinium into organic phase (D2EHPA in kerosene) and using our new modified approach. With the assistance of the hydrodynamic study, plug flow, backflow, axial dispersion, and forward mixing models were established to determine K_od_ × a. Experimental results were employed to validate the predicted results from the mathematical models.

## Models description

Four usual models for studying mass transfer in the liquid–liquid extraction columns are the plug flow model (PFM), axial dispersion model (ADM), backflow model (BFM), and forward mixing model (FMM). The following equations represent the mass balance for each model, describing the transfer of mass from the continuous phase to the dispersed phase.

**Figure 1:**
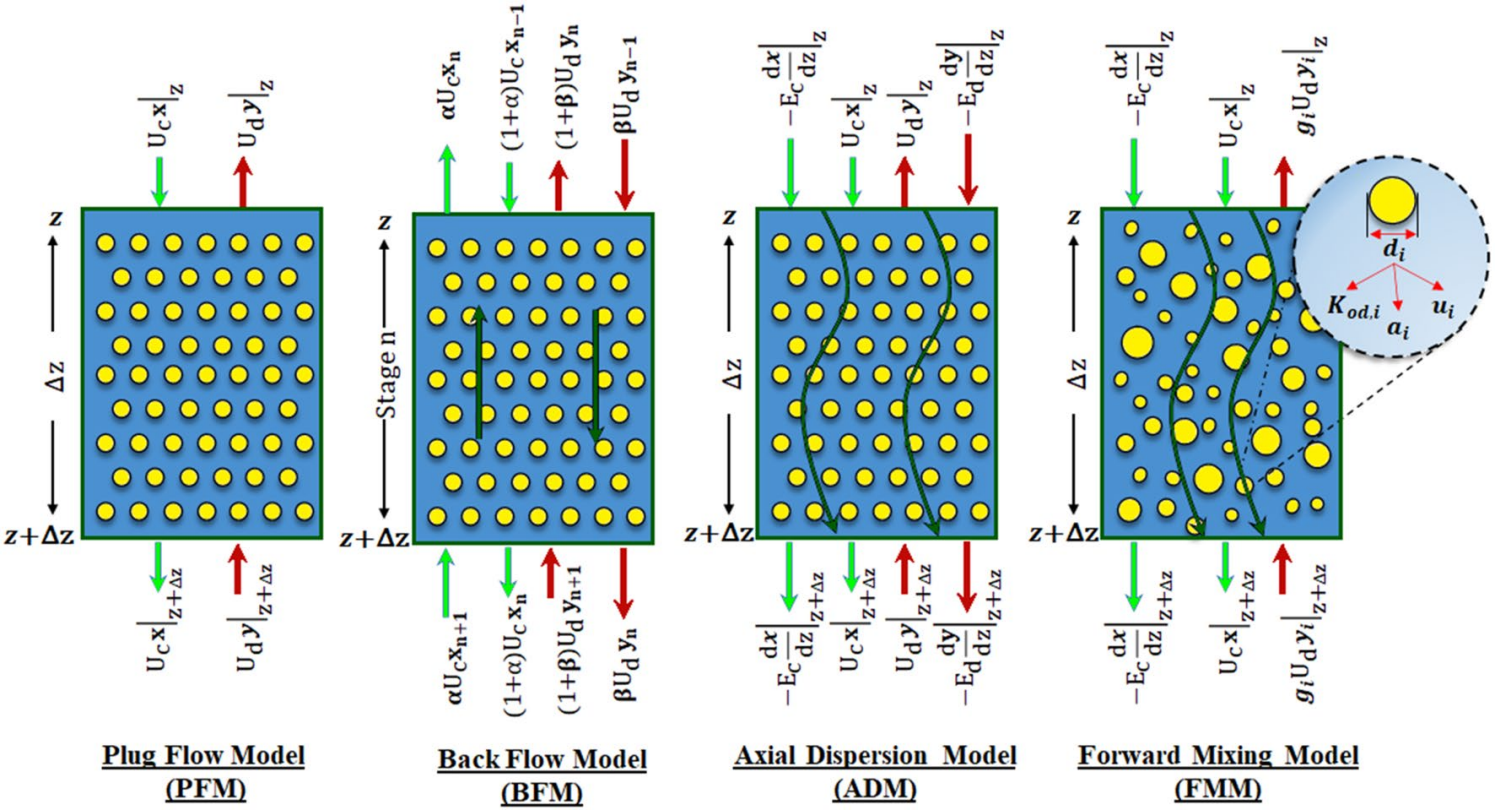
Mass balance over a volumetric element based on the plug flow, back-flow, axial dispersion and forward mixing models.

Both equations for aqueous and organic phases with the PFM model according to Fig. 1 are illustrated as follows^29^:

1 $$\frac{dX}{dZ}+\Omega NT{U}_{od} \left(X-Y\right)=0$$

2 $$\frac{dY}{dZ}+NT{U}_{od}\left(X-Y\right)=0$$

In the above equations, the boundary conditions can be described as follows^29^:

3 $$Z=0\to {X}^{0}={X}^{in}=1$$

4 $$Z=1 \to {Y}^{1}={Y}^{in}=0$$

The BFM model is written stage by stage through the equipment. Two coefficients define the deviation from plug flow for continuous and dispersed phases, α and β, respectively. The backmixing is characterized by backmixing ratio (α, β) of backmixed to net forward flow. The writing of the mass balance equations for both phases according to Fig. 1 is as follows^29^:

5 $$\left(1+\alpha \right){ X}_{n-1}-\left(1+2\alpha \right) {X}_{n}+\alpha { X}_{n+1}-\frac{\Omega NT{U}_{od}}{N}\left({{X}_{n}-Y}_{n}\right)=0$$

6 $$\left(1+\beta \right){ Y}_{n+1}-\left(1+2\beta \right){ Y}_{n}+\beta { Y}_{n-1}+\frac{NT{U}_{od}}{N} ({{X}_{n}-Y}_{n})=0$$

The boundary conditions for this model are illustrated as follows^29^:

7 $$Z=0 \to \left\{\begin{array}{l}{X}_{0}+\alpha ({X}_{0}-{X}_{1})=1\\ {Y}^{0}={Y}_{0}={Y}_{1}\end{array}\right.$$

8 $$Z=1 \to \left\{\begin{array}{l}{X}^{N+1}={X}_{N+1}={X}_{N} \\ {Y}_{N+1}-\beta \left({Y}_{N}-{Y}_{N+1}\right)=0\end{array}\right.$$

The axial dispersion model illustrates the presence of non-uniform flows in both the continuous and dispersed phases. The back mixing of each phase can be characterized by a constant turbulent axial diffusion coefficient, E_c_ or E_d_. The mass balances on the solute in two phases lead to the following equations^29^:

9 $$\frac{dX}{dZ}-\frac{1}{P{e}_{c}}\frac{{d}^{2}X}{d{Z}^{2}}+\Omega NT{U}_{od}\left(X-Y\right)=0$$

10 $$\frac{dY}{dZ}+\frac{1}{P{e}_{d}} \frac{{d}^{2}Y}{d{Z}^{2}}+NT{U}_{od}\left(X-Y\right)=0$$

The four boundary conditions are obtained as follows^29^:

11 $$Z=0 \to \left\{\begin{array}{c}\left(\frac{{U}_{c}}{{E}_{c}}\right) \left(1-{X}^{0}\right)=-{\left.\frac{dX}{dZ}\right|}_{0} \\ {\left.\frac{dY}{dZ}\right|}_{0}=0 \to {Y}^{0}={Y}^{out}\end{array}\right.$$

12 $$Z=1 \to \left\{\begin{array}{c}{\left.\frac{dX}{dZ}\right|}_{1}=0\to {X}^{1}={X}^{out}\\ \left(\frac{{U}_{d}}{{E}_{d}}\right) \left({Y}^{1}\right)=-{\left.\frac{dX}{dZ}\right|}_{1}\end{array}\right.$$

The BFM and ADM models are commonly employed as the preferred options for assessing the effectiveness of mass transfer. The most important assumption is the uniform of drop size. The uneven drop size distribution leads to the deviation of the mass transfer coefficients from the ‘ideal’ condition. The FMM model with the application of drop size distribution is illustrated to overcome the stimulus’s non-ideality and modification. Writing the mass balance equations in a given column volume according to Fig. 1 leads to the following equations, which are^30^:

13 $$\frac{dX}{dZ}-\frac{1}{P{e}_{c}} \frac{{d}^{2}X}{d{Z}^{2}}+\Omega \sum_{i=1}^{N}{ NTU}_{od,i} \left({X-Y}_{i}\right)=0$$

14 $$\frac{d{Y}_{i}}{dZ}+\frac{{NTU}_{od,i}}{{g}_{i}} \left({X-Y}_{i}\right)=0 (i=1, 2,\dots N)$$

The dynamic drop size distribution (g_i_) in the above equation is illustrated as follows^30^:

15 $${g}_{i}=\frac{{f}_{i} {u}_{i}}{\sum_{j=1}^{N}{f}_{j} {u}_{j}}$$

The drop velocity (u_i_) can be calculated using Eq. (16)^30^:

16 $${u}_{i}=\frac{{d}_{i}}{{d}_{43}} {U}_{slip}-\frac{{U}_{c}}{1-\varphi }$$

The boundary conditions related to the inlet and exit concentrations of each phase are obtained as follows^30^:

17 $$Z=0 \to \left\{ \begin{array}{l}\left(\frac{{U}_{c}}{{E}_{c}}\right) (1-{X}^{0})=-{\left.\frac{dX}{dZ}\right|}_{0} \\ \\ {Y}_{i}^{0}={Y}_{i}^{out} \left(i=1, 2,\dots N\right)\end{array}\right.$$

18 $$Z=1 \to \left\{\begin{array}{l}{\left.\frac{dX}{dZ}\right|}_{1}=0 \to {X}^{1}={X}^{out} \\ \genfrac{}{}{0pt}{}{ }{{Y}_{i}^{1}={Y}^{in}=0 \left(i=1, 2,\dots N\right)}\end{array}\right.$$

## Experimental details

**Figure 2:**
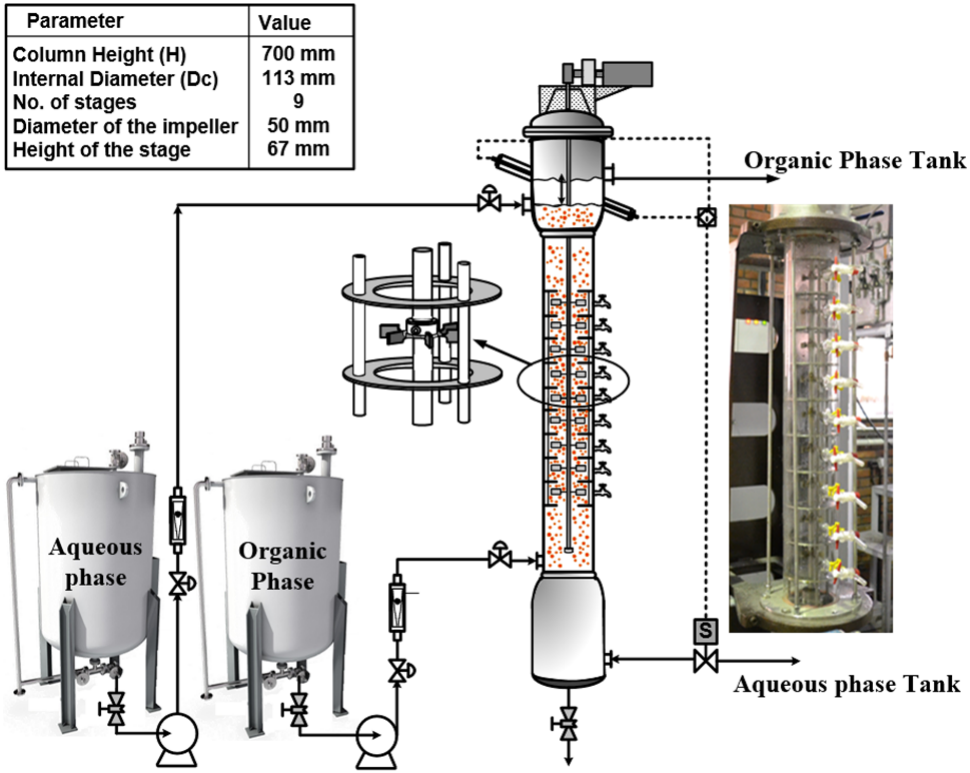
Schematic of pilot plant multi-impeller agitation column.

To study mass transfer models, it is necessary to prepare a series of laboratory data. The basis of the experiments was performed using a multi-impeller agitated column. The experimental system was 0.1 M di-(2-ethylhexyl)phosphoric acid (in kerosene)-samarium and gadolinium ions with 500 ppm concentration (Sm(III), and Gd(III))-nitrate solution with pH ~ 1.5. The organic phase was used as the dispersed phase. Figure 2 displays the experimental arrangement which consists of a glass portion measuring 113 mm in diameter and with an efficient height of 700 mm.

A total of nine number stages were present in the described column. The nine sampling taps were situated in nine stages. Sampling is conducted from every tap located within the operational section of the column during stable circumstances. The samples acquired consist of two distinct phases, namely aqueous and organic. These phases are subsequently separated using a decanter, and the concentrations within the aqueous phase are scrutinized for analysis. The concentrations of Sm(III) and Gd(III) were measured along the column with an inductively coupled plasma mass spectrometry instrument (ICP-MS) to obtain a system concentration profile under steady-state conditions. Sampling was conducted on three separate occasions for each of the nine sampling taps while ensuring steady-state conditions. After determining the element concentration, the average outcomes were utilized for analysis and modeling.

The internal assembly of the glass column is made of SS316. The stirrer was located in the center of each compartment with 6-blade impellers (50 mm O.D) and these impellers were driven by an electric motor. The start-up of the multi-impeller agitated column was far from flooding conditions. Simulations were validated using hydrodynamic parameters, including drop size and holdup measured at various agitation speeds. To determine the average drop size and its distribution, photographs were taken using a digital camera. The calculation of the Sauter mean diameter involved the utilization of the following equation.

19 $${d}_{32}=\frac{\sum_{i=1}^{N}{n}_{i}{d}_{i}^{3}}{\sum_{i=1}^{N}{n}_{i}{d}_{i}^{2}}$$

To ensure the statistical significance of the determined size distributions, a minimum of 1000 drops were examined in each experimental image. By utilizing a reference point in the image such as the thickness of stators, a straightforward proportionate connection could be established between these two values, enabling the determination of the relative actual size of the drops.

To measure the number density and size distribution, the sizes of droplets are divided into several ranges, ± 0.1 mm. The following equation calculates the number of drops:

20 $$pdf=\frac{number\;of\;drops\;of\;classes\;i}{total\;number\;of\;drops}$$

The dispersed phase holdup was obtained using the shutdown method and the measurement of the dispersed phase holdup in the column’s upper settler section. After accumulation in this section, the variation in the interfacial height is used for the determination of φ with the volume of dispersed (V_d_) and continuous (V_c_) phases, as follows:

21 $$\varphi =\frac{{V}_{d}}{{V}_{c}+{V}_{d}}$$

The slip velocity was observed by the following equation:

22 $${U}_{slip}=\frac{{U}_{d}}{\varphi }+\frac{{U}_{c}}{(1-\varphi )}$$

## Results and discussion

**Figure 3:**
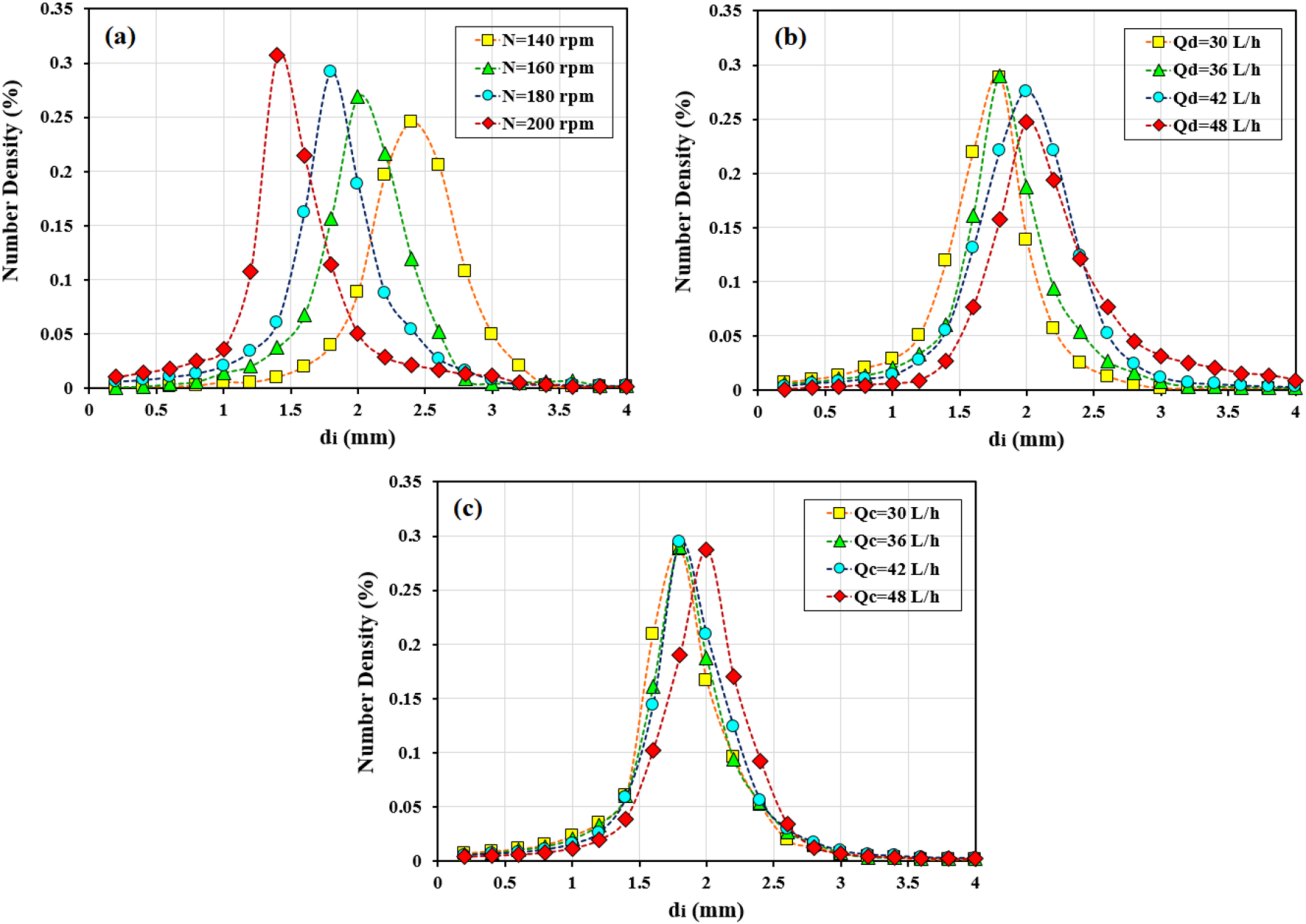
Variation of drop size distribution with the (**a**) agitation speed (Q_d_ = Q_c_ = 36 L/h); (**b**) organic phase flow rate (N = 180 rpm, Q_c_ = 36 L/h); (**c**) aqueous phase flow rate (N = 180 rpm, Q_d_ = 36 L/h).

Before examining the mass transfer performance inside the multi-impeller agitated column, the necessary studies were performed by measuring the droplet sizes, dispersed phase holdup, slip velocity, and extraction efficiency, which are shown in Table 1. The variation in drop size distributions with the operating conditions is shown in Fig. 3. Evaluation of the data in this Table showed that the variation in these parameters strongly depends on the agitation speed. Also, the drop size distribution shows the smaller and narrower sizes with the increments in the agitation speed. The same behavior is observed extracting other ions in the agitation columns in the literature^31–33^.

**Table 1:** Experimental data for extraction of gadolinium and samarium under different operating conditions.

Run No.	Operating Conditions			Hydrodynamic parameters				Extraction efficiency (%)	
	Q_c_ (L/h)	Q_d_ (L/h)	N (rpm)	d_32_ (mm)	ϕ (–)	U_Slip_ (mm/s)	a (m^2^/m^3^)	Gadolinium	Samarium
1	36	36	140	2.44	0.042	24.62	104.06	72.38	47.95
2	36	36	160	2.21	0.052	20.47	139.51	85.89	62.61
3	36	36	180	2.07	0.063	16.78	183.99	89.12	65.95
4	36	36	200	1.89	0.076	14.28	239.89	95.14	76.67
5	36	30	180	1.91	0.054	19.22	170.91	67.35	55.19
6	36	42	180	2.19	0.073	15.01	198.75	91.98	70.17
7	36	48	180	2.29	0.079	14.14	205.91	94.03	72.11
8	30	36	180	2.03	0.06	14.81	178.68	90.34	68.82
9	42	36	180	2.11	0.066	18.57	189.09	69.01	42.93
10	48	36	180	2.14	0.07	20.21	194.92	46.22	28.26

Increasing the agitation speed in the column is associated with a reduction in droplet diameter and droplet breakage. This factor causes an increase in the dispersed phase holdup and a decrease in slip velocity between the droplets. On the other hand, the required interfacial area for the reaction increases with the change of hydrodynamic parameters, and therefore, the process efficiency increases. This factor increases the extraction percentage of gadolinium and samarium ions from 72.38 and 47.95% to 95.14 and 76.67%, respectively.

The increment in the inlet aqueous phase flow rate showed limited effects on the drop sizes, and holdup. The same results for drop size distribution are observed in Fig. 3. The decrease in slip velocity is due to the increase of drag forces between the droplets and the continuous phase. This factor causes the limited motion of droplets and increased residence time. The rate of Sm(III), and Gd(III) extraction with the higher values for Q_c_ decreases because there is not much increase in the interfacial area of mass transfer. Therefore, there is no desire to achieve further extraction. The increase in the holdup in the agitated column occurs with increasing Q_d_; this factor increases droplet sizes due to the increment in coalescence rate (see Fig. 3 for the variation in drop size distribution). The interfacial area for the reaction increases with increasing Q_d_ (from 30 L/h to 42 L/h), which effectively increases the extraction percentage from 55.19% and 67.35–72.11% and 94.03% for samarium and gadolinium ions, respectively.

**Figure 4:**
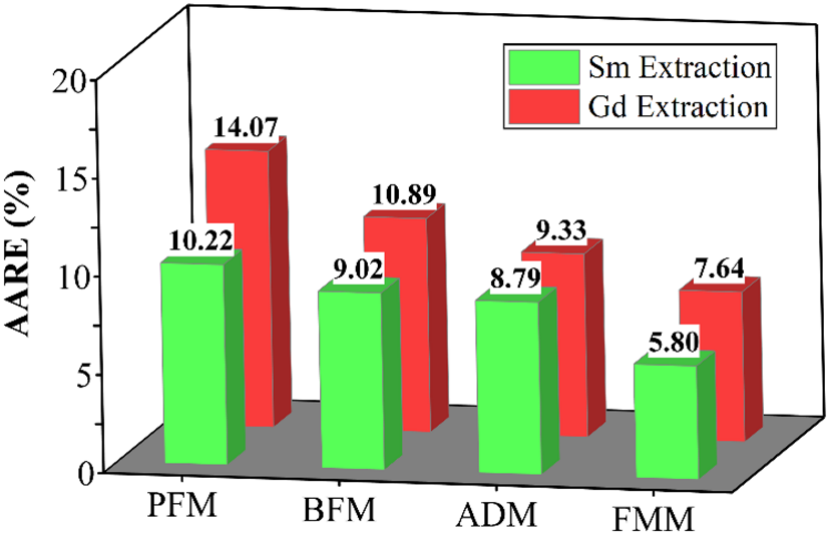
Comparison of the obtained AARE values by different models.

Laboratory data were evaluated to analyze four mass transfer models (PFM, BFM, ADM, FMM) by using Eqs. (1)–(18). Figure 4 displays the average absolute relative error (AARE) for these models. The findings indicate that the forward mixing model is highly suitable for examining the extraction behavior of samarium and gadolinium. In this particular model, the calculation process involves assuming the droplet size distribution and considering the non-uniform behavior within the column. This factor has reduced the model error and is very close to the experimental data values through the curve fitting approach, which agrees with other data in the pulsed and agitated extraction columns^34–36^.

**Figure 5:**
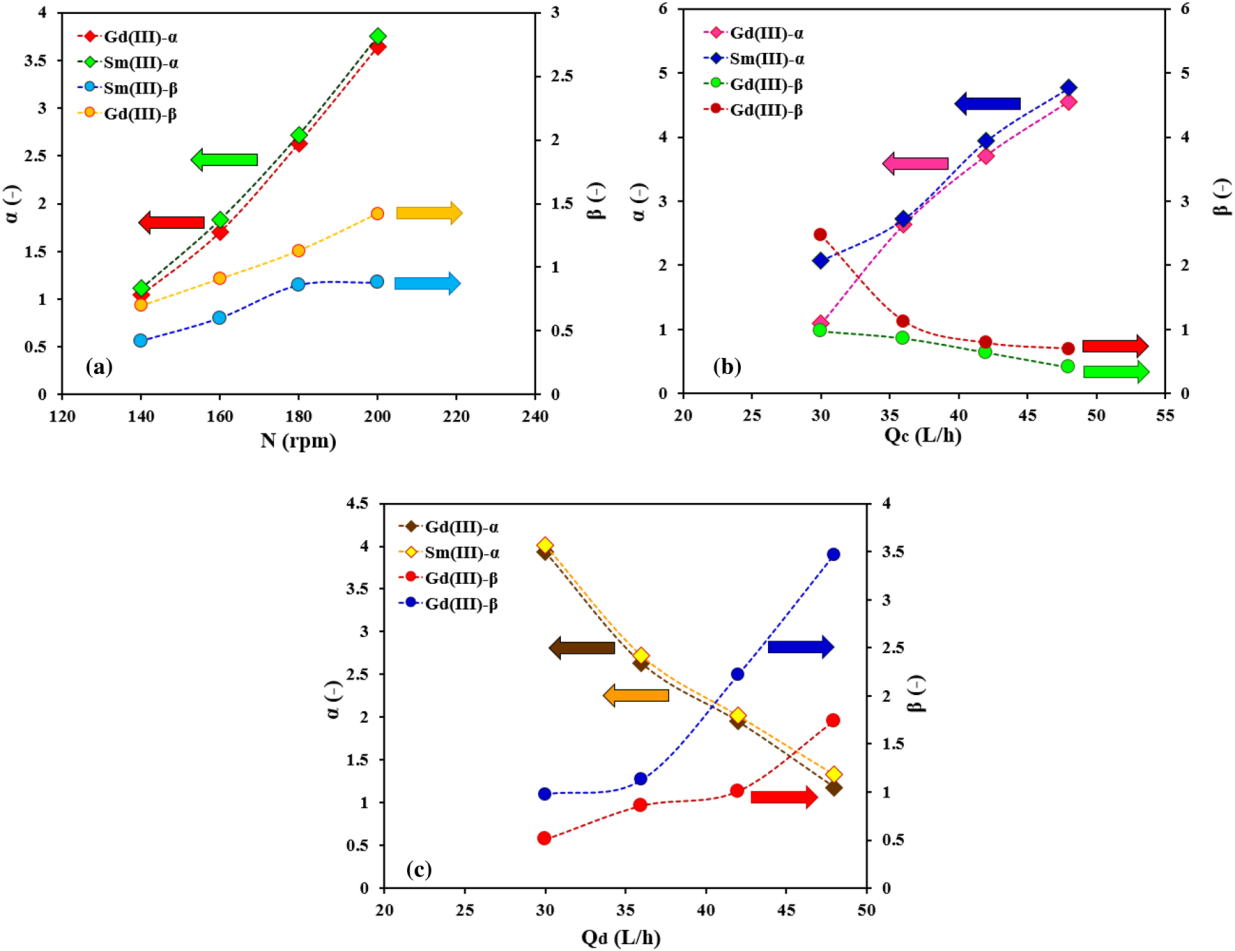
Effects of operating parameters (**a** agitation speed, **b** aqueous phase flow rate, and **c** organic phase flow rate) on the continuous and dispersed phase backflow coefficients.

The described parameters in the backflow model were calculated based on the organic and aqueous side-phases or dispersed and continuous side-phase (α, and β) by analyzing the mass transfer models, the results of which are shown in Fig. 5. The effect of agitation intensity (higher values for rotor speed from 140 to 200 rpm) in a multi-impeller agitated column on α and β parameters is described in Fig. 5a. An increasing trend appears for both parameters. The role of increasing the velocity of the blades in breaking the droplets and increasing the stresses of the droplets inside the column with the internal components causes these coefficients to be directed to more numbers. Increasing the inlet continuous phase flow rate on the α and β coefficients (see in Fig. 5b) reveals the increasing trend for α values and the decreasing trend for β values. The same results are observed for these coefficients with the higher values for Q_d_ from 30 to 42 L/h (see in Fig. 5c).

The coefficients of the axial dispersion model (E_c_, E_d_ for continuous and dispersed phases, respectively) by changing the operating parameters are given in Table 2. The results of this table showed that the trend of changes is similar to the α, and β coefficients in the backflow model. Among operating parameters, the flow rate of the continuous phase has the most minor influence, and agitation speed has the maximum impact on the axial dispersion coefficients. The size distribution in the forward mixing model helps to reduce non-uniform current deviations and creates lower numbers for the axial dispersion coefficients of aqueous side-phase.

**Table 2:** Axial dispersion coefficients by using ADM and FMM models.

Run No.	Operating conditions			ADM model		FMM model
	Q_c_ (L/h)	Q_d_ (L/h)	N (rpm)	E_c_ (× 10^5^ m/s)	E_d_ (× 10^5^ m/s)	E_c_ (× 10^5^ m/s)
1	36	36	140	5.39	1.04	5.33
2	36	36	160	7.54	1.50	7.49
3	36	36	180	10.70	2.48	10.64
4	36	36	200	13.84	2.91	13.77
5	36	30	180	12.11	2.07	12.05
6	36	42	180	8.30	2.75	8.24
7	36	48	180	5.90	3.36	5.83
8	30	36	180	4.34	3.67	4.30
9	42	36	180	14.55	1.74	14.48
10	48	36	180	17.09	1.02	16.99

**Figure 6:**
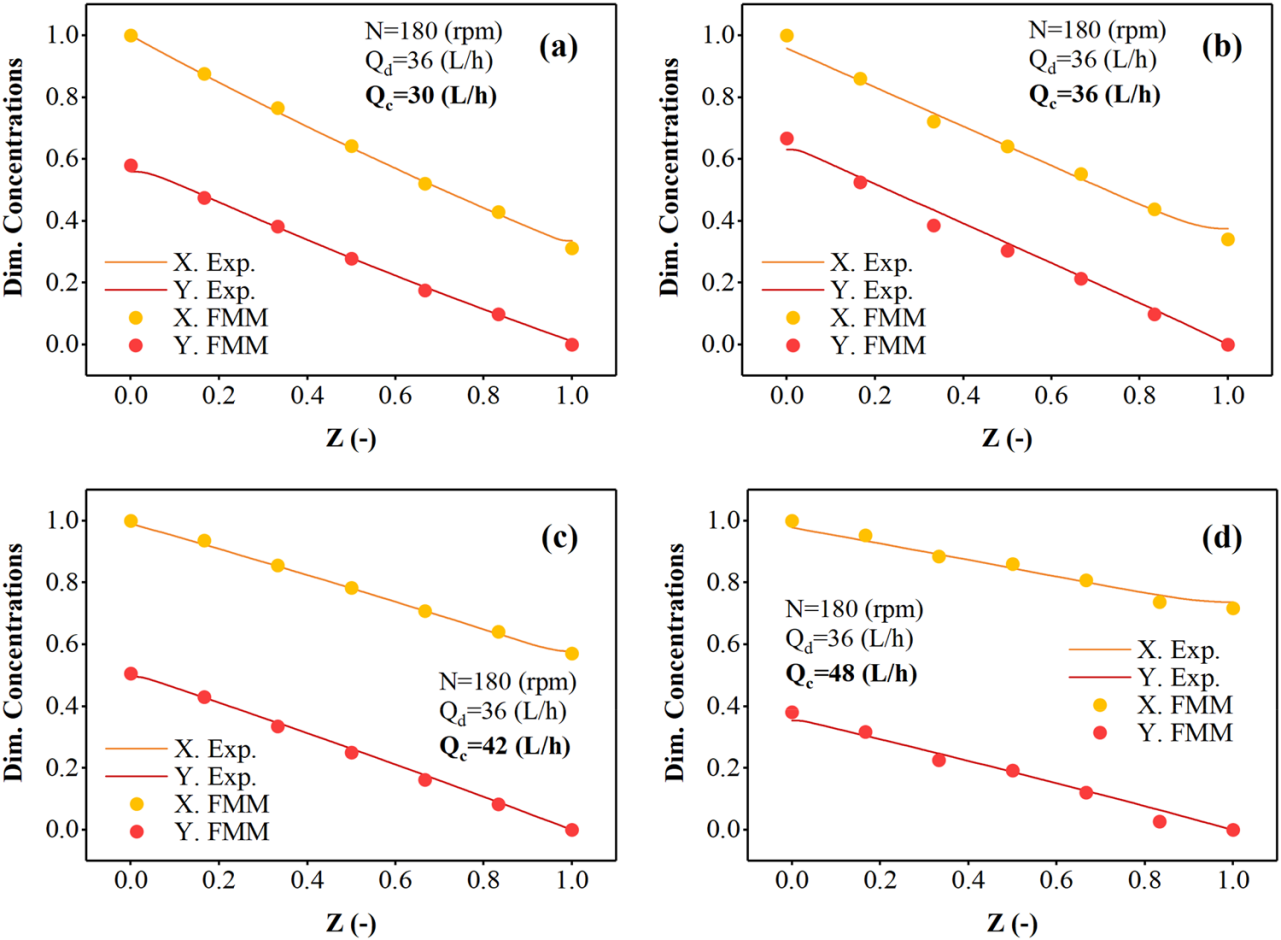
The effect of aqueous phase flow rate on the Sm(III) concentration profiles in continuous and dispersed phases.

**Figure 7:**
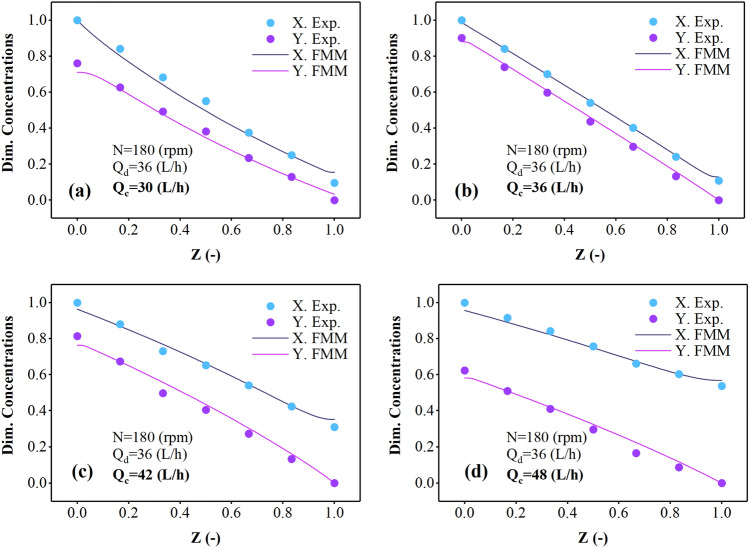
The effect of aqueous phase flow rate on the Gd(III) concentration profiles in continuous and dispersed phases.

Figures 6 and 7 illustrate the alterations in the concentration profile of the dispersed and continuous phases within the multi-impeller agitated column as the flow rate of the continuous phase increases, specifically focusing on samarium and gadolinium ions. The results in this figure showed that the two curves become more distant with increasing Q_c_. Therefore, the increase in the flow of the aqueous phase does not help extract more ions and has a negative effect. Reducing the required time to react and penetrate is the main reason for this trend. The gap between the lines in the curve depicting the variations in samarium concentration is larger than the gap observed in the curve representing the changes in gadolinium concentration. Gadolinium ions appear stronger in extraction and have a greater tendency to react with the D2EHPA extractant and escape to the organic phase, creating a higher extraction percentage.

**Figure 8:**
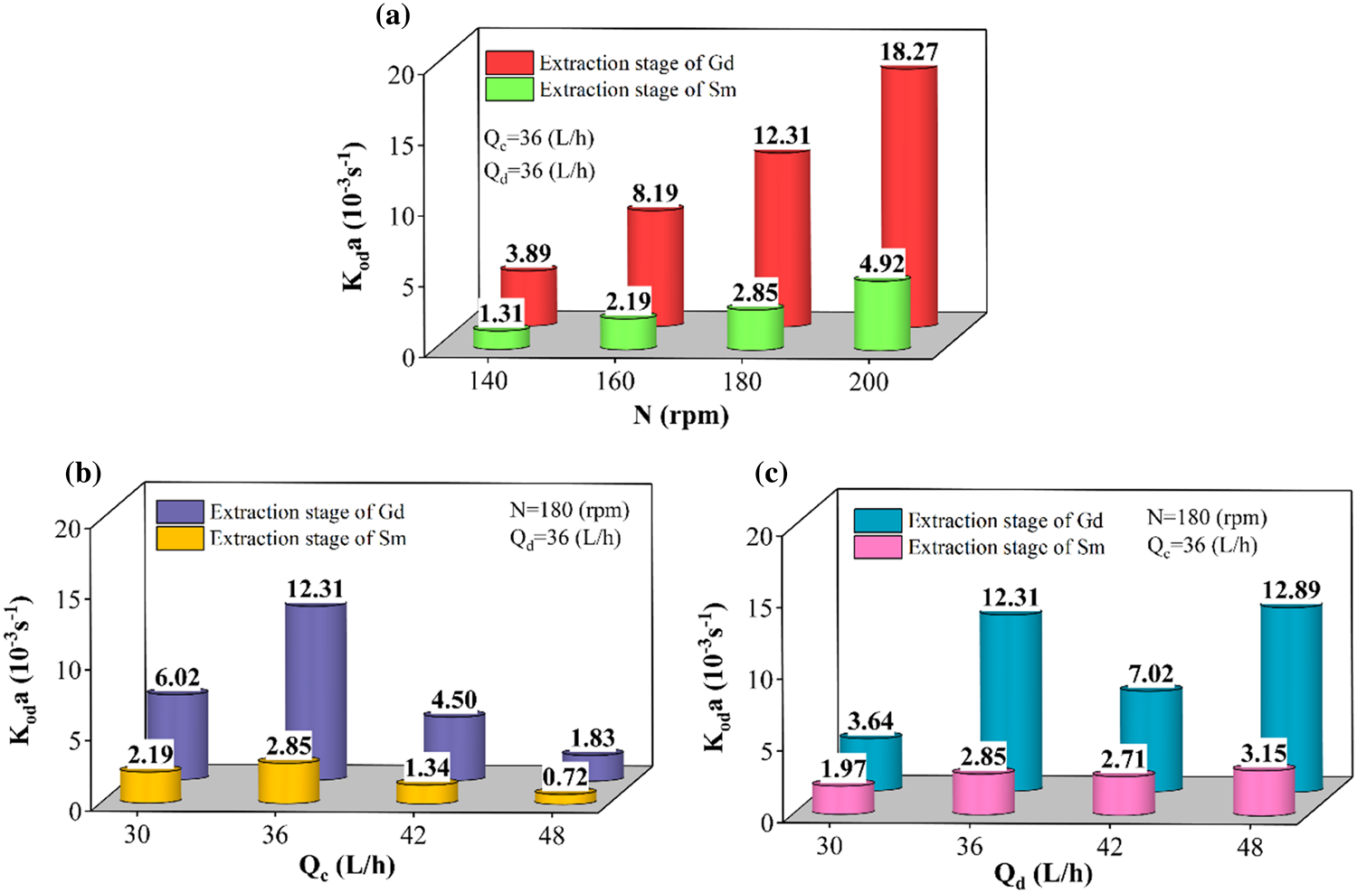
The influence of operating parameters on the volumetric overall mass transfer coefficient obtained by forward mixing model.

The study of mass transfer performance in the multi-impeller agitated column is shown in Fig. 8 by changing the volumetric overall mass transfer coefficients based on the operating parameters. The increase in mass transfer performance with the higher agitation speed, the decrease with the higher values for Q_c_, and their growth with Q_d_ are shown in Fig. 8a–c, respectively. The agitation speed is one of the critical operating parameters for this column. It has a significant effect on dispersed phase holdup, drop sizes, and interfacial area during a reaction.

Increasing the surface area for reaction causes the extraction system in this column to move towards the maximum extraction percentage. It increases the tendency to transfer more rare earth elements (Sm(III), and Gd(III) ions) to the organic phase.

## Conclusion

Four different mass transfer models (PFM, BFM, ADM, and FMM) are introduced in order to assess the efficiency of mass transfer in the multi-impeller agitated column. Droplets of the dispersed phase are assumed to have a constant and uniform size in the PFM, BFM, and ADM models. The size distribution of drops was utilized in the forward mixing model. The mathematical models used to estimate mass transfer coefficients, backflow, and axial dispersion coefficients for both phases using the curve fitting approach. The obtained concentration profile from various models compares well with the experimental values. The FMM model is much better than other models to estimate the concentration profile with the average absolute relative error being less than 8% when estimated values are compared with experimental values. Among operating parameters, the inlet flow rate of the continuous phase has the most negligible influence, and agitation speed has the maximum impact on the mass transfer coefficients, axial dispersion, and backflow coefficients. This work shows that FMM can successfully model the prediction of the performance of the multi-impeller agitated column to extract samarium and gadolinium ions.

## Data Availability

The datasets used and/or analyzed during the current study are available from the corresponding author on reasonable request.

## List of symbols

a: Specific surface area per unit volume (6φ/d_32_) (1/m)
d: Drop diameter (m or mm)
d_32_: Sauter mean drop diameter (mm)
d_43_: Mean diameter (m)
E: Axial dispersion coefficient (cm^2^/s)
*f*: Volumetric drops size distribution (–)
g: Dynamic drop size distribution (–)
H: Height of column (m)
K: Overall mass transfer coefficient (m/s)
N: Agitator speed (rpm)
n: Number of drops (–)
Q: Volume flow rate (L/h)
S: Cross-sectional area of the column (m^2^)
t: Residence time of drops (s)
u: Drop velocity (m/s)
U: Superficial velocity (Q/S) (m/s)
U_slip_: Slip velocity (U_c_/(1 − φ) + U_d_/φ) (m/s)
V: Volume of phases (m^3^)
x: Mass fraction of solute in continuous phase (–)
y: Mass fraction of solute in dispersed phase (–)
z: Height variable (m)

## Dimensionless symbols

‘NTU’: Number of transfer units (K_od_aH/U_d_)
p: Slope of the concentration equilibrium equation (y = p*x + q)
Pe: Peclet number (HU/E)
X: Continuous phase concentration ((x − x^*^_out_)/(x_in_ − x^*^_out_))
Y: Dispersed phase concentration ((y − y_in_)/(y^*^_out_ − y_in_))
Z: Height (Z = z/H)
Ω: Constant (pU_d_ρ_d_/U_c_ρ_c_)

## Greek symbols

α: Backflow coefficient of dispersed side-phase (–)
β: Backflow coefficient of continuous side-phase (–)
μ: Viscosity (kg/m s)
ρ: Density (kg/m^3^)
σ: Interfacial tension between two phases (N/m)
φ: Dispersed phase holdup (–)

## Subscripts/superscripts

*: Equilibrium concentration
c: Continuous phase
d: Dispersed phase
i: Drop class
in: Inlet to the column
j: Cross-section
out: Outlet from the column

## Abbreviations

AARE: Average absolute relative error
ADM: Axial dispersion model
BFM: Back flow model
FMM: Forward mixing model
